# A Systematic Review of Phytochemistry, Nutritional Composition, and Pharmacologic Application of Species of the Genus *Viola* in Noncommunicable Diseases (NCDs)

**DOI:** 10.1155/2023/5406039

**Published:** 2023-10-31

**Authors:** Gaber El-Saber Batiha, Halimat Yusuf Lukman, Hazem M. Shaheen, Lamiaa Wasef, Amin A. Hafiz, Carlos Adam Conte-Junior, Ammar Al-Farga, Moses V. M. Chamba, Bashir Lawal

**Affiliations:** ^1^Department of Pharmacology and Therapeutics, Faculty of Veterinary Medicine, Damanhour University, Damanhour 22511, AlBeheira, Egypt; ^2^Department of Chemical Sciences, Biochemistry Unit, College of Natural and Applied Sciences, Summit University Offa, Offa PMB 4412, Nigeria; ^3^Department of Clinical Nutrition, Faculty of Applied Medical Sciences, Umm Al-Qura University, Mecca, Saudi Arabia; ^4^Laboratory of Advanced Analysis in Biochemistry and Molecular Biology (LAABBM), Department of Biochemistry, Federal University of Rio de Janeiro (UFRJ), Rio de Janeiro RJ 21941-909, Brazil; ^5^Biochemistry Department, Faculty of Science, University of Jeddah, Jeddah 21577, Saudi Arabia; ^6^Department of Physics and Biochemical Sciences, Malawi University of Business and Applied Sciences, Private Bag 303, Chichiri, Blantyre 3, Malawi; ^7^Faculty of Medical Science, New Gate University, Minna, Nigeria

## Abstract

*Viola* L. is the largest genus of the Violaceae family with more than 500 species across the globe. The present extensive literature survey revealed *Viola* species to be a group of important nutritional and medicinal plants used for the ethnomedicinal treatment of noncommunicable diseases (NCDs) such as diabetes, asthma, lung diseases, and fatigue. Many plant species of this genus have also received scientific validation of their pharmacological activities including neuroprotective, immunomodulatory, anticancer, antihypertensive, antidyslipidemic, analgesic, antipyretic, diuretic, anti-inflammatory, anthelmintic, and antioxidant. *Viola* is highly rich in different natural products some of which have been isolated and identified in the past few decades; these include flavonoids terpenoids and phenylpropanoids of different pharmacological activities. The pharmacokinetics and clinical studies on this genus are lacking, and the present review is aimed at summarizing the current understanding of the ethnopharmacology, phytochemistry, nutritional composition, and pharmacological profile of medicinal plants from the *Viola* genus to reveal its therapeutic potentials, gaps, and subsequently open a new window for future pharmacological research.

## 1. Introduction

Medicinal plants have been explored from time immemorial as a source of healing agents against several diseases and have been subjected to scientific analysis for the validation of their efficacy against several human diseases [1–13]. The family Violaceae comprises about 500 species distributed across 20 genera [14]. The plant is popular in the northern part of the world as Zihua Diding [15] and is used traditionally by ancient Chinese against furuncles, carbuncles, and boils [16]. Different species of *Viola* are widely distributed in different geographical locations; seventeen species of the plant are identified in Pakistan which include *Viola biflora, Viola canescens, Viola odorata, Viola tricolor,* and *Viola cinerea* [17]. *Viola biflora* is also widely distributed in Europe, Central Asia, and India [18], and it is distributed as *Viola canescens* in India, Nepal, and Bhutan, as *Viola cinerea* in Iran and Oman, as *Viola odorata* in India, Iran, Afghanistan, and Iraq [19], as *Viola tricolor* in the Mediterranean region, Caucasian, Europe, Asia, America, and Australia [20], as *Viola diffusa* in subtropical Himalaya spreading from Nepal to Mishmi (3000–5000 fit of khasia hills) and China [21], and as *Viola patrinii* in the Himalayas, Eastern and Western Ghats, China, and Japan [21, 22].

This genus has been used by traditional healers for the management and treatment of several human diseases including infectious diseases, diabetes, asthma, lung diseases, cough, fatigue, and several other diseases [23]. Notably, some of these traditional uses have been validated. However, there is paucity of information on the current understanding of the pharmacokinetics and clinical studies of this genus. Thus, this review seeks to gather information on the chemical constituents and therapeutic activities of this genus in NCDs which will subsequently open a new window for future pharmacological research.

## 2. Methodology

Extensive and comprehensive literature search was conducted using about one hundred and ninety (190) articles obtained via electronic scientific databases such as Google, Google Scholar, PubMed, Science Direct, and Web of Science as well as journals obtained through citations or directly from their website. The keywords used during the search included *Viola* distribution, morphology, ethnobotanical, phytochemistry, nutritional composition, toxicology, and pharmacological activities.

## 3. Morphology

**Figure 1:**
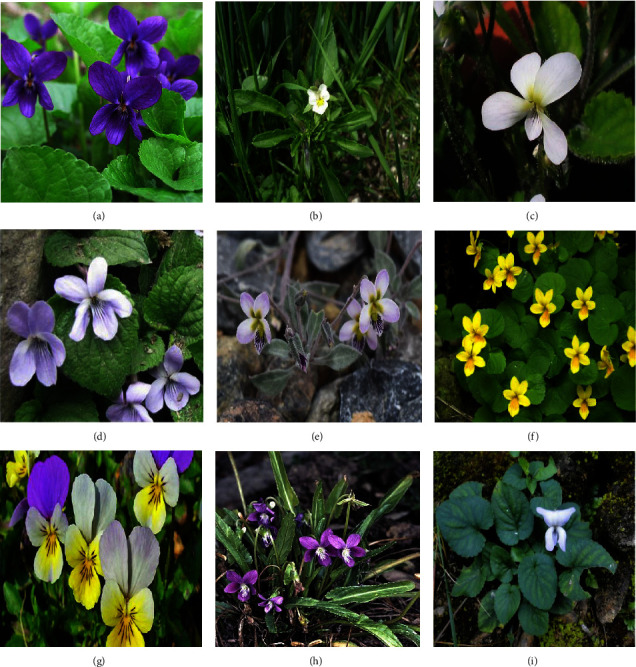
Morphological features of *Viola* genus. (a) *V. odorata.* (b) *V. arvensis*. (c) *V. diffusa.* (d) *V. pilosa.* (e) *V. cinerea.* (f) *V. biflora.* (g) *V. tricolor.* (h) *V. betonicifolia.* (i) *V. canescens*.

The species of this genus exist mainly as biennial or perennial herbs in the tropical, subtropical, and temperate regions; however, they occasionally occur as subshrubs and rhizomatous. The general morphology of *Viola* revealed that the genus have a short stem with or without rhizomes and are capable of growing yearly or within months, the shape of the leaves ranging from being ovate-triangular to being cordated, serrated, crenated, stalked, and could be having wings [24]. They can also be stipules, lanceolate-ovate, entire dentate, or fimbriate. *Viola* flower has five sepals and five spurs forming petals, zygomorphic, peduncle bracteolate, and has cone-shaped anthers that cover the ovary, short, broad, and distinct filaments that form a corolla spur while the ovary sessile has a thickened top with curved bottom style and a stigma that could be lobed, straight, or beaked. Their seeds are round, ovate, smooth, and shiny with a 3-valved capsule shaped fruit [25]. However, *Viola* has numerous species (Figure 1) of varying morphology; some of the species are described as follows:

 
*Viola odorata* L.: the morphology of *Viola odorata* is of heart shape, its leaves are simple toothed arranged at the base, perennial herb stock short, sometimes branched, knotted with the remains of old leaves stalks and stipules, and usually emitting creeping runners or scions. The plant has a thick and scaly underground stem, with rooting runners. It possesses heart-shaped leaves with scalloped or slightly serrated edges that are dark green, radical tufts, broadly cordate, round at the top, smooth or sometimes downy underneath, and grow in a rosette at the base of the plant [26], while the flowers are either deep purple, blue, pinkish, or yellowish white [26, 27].
 
*Viola cinerea* Boiss: Annual or perennial herb, erect or ascending, up to 20 cm tall; stems glabrous to densely pubescent. Leaves with lamina narrowly ovate or narrowly lanceolate, up to 30 × 14 mm [28].
 
*Viola diffusa* Gingins: It has a slightly 2-lobed stigma, long stolon with rosulate leaves at the apex, purplish flowers, a short corolla spur, and pubescent leaf blades [29].
 
*Viola biflora* Linn: this species is glabrous or pubescent. The stem is usually erect, 7.5–25 cm. leaves 2-3, kidney shaped, 2–2.5 cm. across crinate, stipules, ovate, or oblong. Flowers 1 or 2, with the same stalk, pale yellow, lower petals streaked to black, spur very short, and stigma two lobbed [30].
 
*Viola patrinii* DC: it is glabrous or pubescent with short stems, tufted leaves that are triangular, usually narrowly elongated, 3.8–6.3 by 1.3–3.8 cm long. The flowers are usually dark lilac and often scented [31].
 
*Viola tricolor* L.: the style and stigma have visible hollow, hairs, and papillae on stigma lip. It has a filamentless stamens, visible anther appendix, and lateral anther hairs [32].
 
*Viola betonicifolia* Smith: it is a perennial herb, and its height is from 8 to 20 cm. Distinguishing characteristics of this species are that it is longer in length, slim, has arrow-shaped leaves, which is usually enlarged from the base, possesses no stem, and is 6 cm (in length with a V-shaped sinus at the base). The length of lamina is mostly 1–8 cm with a width of 5–25 mm. The leaf margins of this plant are entirely or marginally serrate. Linear stipules are present which are fused with the petiole and may be entire or laciniate. The sepals are 3.5–7 mm long. The color of the petals is violet but can also be whitish. Petals are 8.5–14 mm long. The plant taste is a little bit piquant and has a spicy fragrance [20]. The seeds are rounded-ovate, smooth, and shiny [25]. The flowers of this plant are shadowed through small pale brown pods with tiny blackish seeds. The roots and rhizome are slender, unbranched, and short, respectively [23].
 
*Viola canescens* Wall: it is subglabrous or hairy, almost perennial prostrate herb. Its roots are cylindrical, profusely branched as well as long. Leaves are broad, ovate, and reniform and may be cordate to acute tips. Leaf margins are serrate or crenate. The length of the leaf petiole is almost twice the length of the lamina. Leaves are pubescent and stipules are freely present. The approximate width and length of lanceolate leaves are 5.0–20.0 and 1.0–3.0 mm. At the base, they are reddish. Stems are absent. Flowers are deliberate with the size of 1.0–1.8 cm approximately. The colors of its flowers range from pale violet to violet and often almost white. Lateral spur is almost 10 mm in length. Sepals are 5 in number and are almost 2 mm wide, dentate near its base. Petals are up to 15 mm long and their width is 4.0 mm. Other characteristics of the petals include obtuse and obovate tips, and the two upper petals are wedge-shaped, and the two lateral petals are narrow and hairy at their base and dark clear streaks are found on them. Himalayan white violet's style is club-shaped and the ovary shape is ovate with hairs [33].
 
*Viola pilosa* Blume: this specie is peculiar within V. ser. Serpentes (W. Becker) steen is a group of about ten species mainly distributed in the southern and southeastern part of Asia, is characterized by an acaulescent stoloniferous habit [34].

### 3.1. Chemical Constituents

*Viola* is highly rich in different natural products out of which about 200 compounds have been isolated and identified in the past few decades; these include flavonoids, coumarins, alkaloids, triterpenoids, saponins, anthocyanins, phenols, tannins, phytosterols terpenoids, lignans, sesquiterpenes, cyclotides, and phenyl propanoids of different pharmacological activities such as antibacterial, antioxidant, anti-inflammatory, and antihypertensive effects among others [35]. This is as evidenced by studies demonstrating that the ethanolic and methanolic extract tested positive to different phytochemicals.  Table 1 shows the chemical constituents of six species of *Viola*.

### 3.2. Essential Oils of *Viola*

Essential oils are compounds of known antioxidant, antibacterial, and antiseptic properties which can be extracted from the plant by hydrodistillation solvent extraction method complemented with gas chromatography mass spectrometer analysis. Analysis of a species of *Viola* essential oils revealed the presence of mainly butyl-2-ethylhexylphthalate and 5,6,7,7a-tetrahydro-4,4,7a-trimethyl-2(4H)-benzofuranone. Essential oils are generally used in the perfumery industry [36], while the pigment extract from the flowers are used for litmus testing strips and in making excellent ground cover [37].

Several reports have documented the compounds isolated and identified in the essential oils of *Viola. V. odorata* contained the highest number of isolated compounds while lower compounds were identified from *V. thianschanica.* The sesquiterpenes and aliphatic compounds formed the predominantly isolated compounds in the various species but were not isolated from *V. thianschanica* species. These constituents are presumably due to the different location and/or time of collection of the plant. Bioactive compounds identified in *Viola* are shown in Table 2.

## 4. Traditional Uses

There is a high demand of medicinal plants for improved quality of life [38, 39]. The biodynamic compounds found in medicinal plants are of important analeptic value [43, 45] rich sources of fuel, fodder, and timber. Various side effects accompany different kinds of commercial drugs which have led to the increase in the use of safe, cheap, and effective herbal remedies for different ailments [44]. The folk people of Pakistan (in Swat, Hazara, and Dir districts), India, Nepal, Sri Lanka, China, Malaysia, and Australia used *V. betonicifolia* in the treatment of pyrexia, astringent, cancer, purgative, and neurological disorders [46], skin, sinusitis, blood disorders, and pharyngitis [47], the roots in the treatment of respiratory and kidney diseases, flowers for respiratory diseases such as asthma, cough, and colds and the leaves for boils [40].

*Viola tricolor* has been used traditionally against skin disorders. Heartsease (*Viola tricolor* L.) has been used for centuries in Europe against inflammatory lung and skin disorders and psoriasis [41, 42]. Several handbooks of phytotherapy have reported traditional use of *Viola* [52] such as in the German commission E Monograph (phytotherapy and herbal substances) of the German Federal Institute for Drugs and Medical Devices [48] and in the Pharmacopoeia [49]. The ethnomedicinal uses of *Viola* are shown in Table 3.

### 4.1. Nutritional Composition

The macronutrients and micronutrients required for a healthy living can be obtained via the consumption of plants. Over 30, 000 wide edible plants (WEPs) have been identified around the globe as potential nutrient supply, one of them is the *Viola* species [50]. The edible flowers *Viola* species are used in cuisines, desserts, and beverages majorly due to their different colors, shapes, and flavors [51]. Nutritional and chemical characterizations of edible petals and corresponding infusions of *Viola* species are used for valorization as new food ingredients. Although, some of the species are not currently used in food and thus considered unconventional food plants; however, their rich nutritional and bioactive content make them display a positive effect on health [53, 54]. Previous study reported that 100 g of dry sample of *Viola x Wittrockiana* contained a total carbohydrate, crude protein, fat, and ash content of 80.27 g, 10.14 g 1.67 g, and 7.92 g, respectively, with a total energy value of 376.67 kcal [55].

*Viola betonicifolia* plant powder was reported to be a potential excellent source of nutrient and food supplement as it contained fats, proteins, carbohydrates, fiber, and vitamin C [23, 67]. Fernandes et al. [68] reported different nutritional content of three different varieties of *Viola x Wittrockiana* colors (white, red, and yellow) at different developmental stages. They found that a 100 g fresh weight of white and yellow species had a higher protein content of more than 2.00 g, the red specie had the highest carbohydrate content of 8.0 g while the fatty acids; linoleic acid (more predominant), palmitic, and linolenic acid were present in all [68]. During the flowering stage, an increase in the nutrient and bioactive content was observed in the white and yellow species; however, that of the red species remained the same but displayed a higher total carotenoid and anthocyanin content. The study hence suggests that the plant can be used to improve the quality of food [68].

## 5. Pharmacological Activities

*Viola* has numerous pharmacologically activities which have been scientifically proven by the acclaimed traditional use against microbial infections, hypertensive, HIV, pyrexial, inflammatory, plasmodial, diuretic, cancer, and so on [20, 26]. These activities can be attributed to the phytochemical constituents that can be harvested and utilized for drug development for both clinical and commercial purposes.

### 5.1. Antioxidant Activity

Antioxidants are usually of plant origin; they help neutralize, lessen, or scavenge the deleterious effect of free radicals in disease conditions. The phytochemicals contain compounds that prevent free radical production or activation of detoxifying protein [69]. Report shows the *in vitro* effect of all extracts in antioxidant study [70]. The aqueous extract of the *Viola odorata* flower showed the antioxidant potential of 2,2-diphenyl-1-picrylhydrazyl radical [69]. Both chloroform and methanolic extracts of *V. odorata* showed antioxidant activity by stalling the bleaching assays of *β*-carotene/linoleic acid and 2,2-diphenyl-1-pycril hydrazyl (DPPH). Antioxidant activity was not observed in the essential oil [36]. Moreover, the antioxidant activity of *V. odorata* extracts (DCM, ethyl acetate, ethanolic, and aqueous) tested by DPPH scavenging activity, metal chelating capacity, ferric, and phosphomolybdenum-reducing antioxidant potential displayed low to moderate activities [71].

### 5.2. Laxative and Diuretic Activities

Crude ethanolic extract *of V. canescens* leaves was demonstrated to possess laxative activity in BALB/c mice using the charcoal meal paradigm in a dose-dependent and methanolic extract of *Viola serpense*. Alcoholic and aqueous extracts at 200 mg/kg and 400 mg/kg, respectively, have significant laxative effects [72].

The diuretic study of n-hexane, butanol, methanolic, and aqueous extracts at doses of 200 and 400 mg/kg body weight showed the presence of flavonoids in the different extracts. The aqueous extract possesses diuretic property at 400 mg/kg which is indicated by increased potassium and sodium ion levels in urine production. The highest dose exhibited good results in all extracts in the first 5 hours and after 24 hours *n*-hexane and methanolic extracts showed the best results. Flavonoid glycosides are reported to have diuretic activity and may be assumed for the extracts' diuretic activity [73].

### 5.3. Anticoagulant Activity

Dietary rich anticoagulants or phytochemicals have been scientifically reported to decrease thromboembolic disease occurrence, thus potentiating *Viola* as a good anticoagulant [74, 75]. *Viola yedoensis* Makino is used traditionally by the Chinese to treat furuncle, carbuncles, release toxic heat, anti-inflammatory, and as antisnake venom [76]. Traditional Chinese herbal medicine used it against *Helicobacter pyloria* [77] and HIV [78]. The anticoagulant activities of three (3) new isolates of dicoumarin; dimeresculetin, euphorbetin, and esculetin identified from *V. yedoensis* Makino may be as a result of activation of partial thromboplastin time (aPTT), prothrombin time (PT), and thrombin time, potentiating *Viola yedoensis* Makino as a potential anticoagulating drug [79].

### 5.4. Anticancer Activity

Flowers, leaves, and stems of *V. canescens* were reported as a pharmacological tool for antitumor due to the presence of cycloviolacin. Cycloviolacin is a cyclotide in plants with antitumor properties that act by creating pores on the cell membrane for entry of substances that could then kill the cancer cells. Cyclotoxic cyclotides are chemosensitive contrary to drug-resistant breast cancer [80, 81]. Cyclotides are cyclopeptides of unusual structure and various biological activities [82]. Vigno 5 is a natural cyclopeptide identified from *Viola ignobilis* [83, 84] demonstrated inhibitory effect on growing cervical cancer cells. It was also reported to significantly elevate caspase-3 expression, reduced antiapoptotic Bcl-2, and increased proapoptotic Bax, leading to the loss of MMP and the release of cytochrome C from the mitochondria membrane in Hela cells, which explains the membrane permeability effect [85]. Activated Bax is translocated as well as integrated into mitochondrial membranes in apoptotic cells [86, 87]. Mitochondrial damage, depolarization, MMP collapse, cytochrome C release, and caspasse-3 activation through either homologous dimerization or the promotion of mPTP formation in the inner and outer mitochondrial membranes may be caused by the overexpression of Bax and low expression of Bcl-2 [88]. IC_50_ values of 820 and 1850 *μ*M of circulin B and cyclopsychotride A, respectively, were reportedly cytotoxic in mouse fibroblasts [89] while varv A and F from *Viola arvensis* and cycloviolacin O_2_ from *Viola odorata* showed antitumor/cytotoxic activity. These antitumoric activities could be leveraged on to produce novel clinical antitumor drugs [80]. Further details on the cytotoxic activities of cyclotides are reported by Göransson et al. [90].

Research is still ongoing on the use of multipurpose anticancer drugs that work by creating pores on cancer cell membranes and allowing the entry of another drug that will act on the cell [91]. This was demonstrated by using doxorubicin drug-resistant breast cancer cells treated with cycloviolacin. Three novel cyclotides from *Psychotria leptothrysa* Miq. var. *longicarpa* Val (Rubiaceae), namely, psyle A, C, and E have been identified [91], although other rubiaceous genera contain cyclotides [92, 93] reports of possible cytotoxicity and chemo sensitization of cyclotides from *Psychotria* are lacking. Cycloviolacin O_2_ isolates exhibited cytotoxic effect [80], due to their chemical components and structure [94, 95]. Cyclotide A and kalata B1 have been studied in different works to demonstrate its chemotherapeutic activity [96]. Membrane disruption is a mechanism of cyclotide cytotoxicity evident by liposomal and whole tumor cells leakage [97], while kalata B1 acts like a channel through the membrane [98]. NMR analysis revealed the hydrophobical binding of cyclotides to surface membranes [99]. These reports altogether indicate that the primary structure of cyclotides and changes in membrane composition influence their bioactivity and membrane affinities [100]. Breast cancer cell lines have been studied to establish the antitumor activities of cyclotides. *In vivo* toxicity and antitumor studies of cycloviolacin has also been described [101].

### 5.5. Antihypertensive and Antidyslipidemic Activities

Anaesthetized rats administered hydromethanolic extract of *Viola odorata*dose-dependently caused a reduction in blood pressure [102]. Hydromethanol extract of common *Viola* leaves has been demonstrated for antihypertensive and vasorelaxatory effects *in vivo* studies. Total cholesterol, low‐density lipoprotein, and atherogenic index were reduced upon treatment with the extract in diet-induced atherogenic rats. There was a significant increase and decrease in high‐density lipoprotein, body weight, no significant difference in glucose, and triglyceride levels, a significant reduction in body weight was observed. The efficacy of this extract was confirmed by the improvement in hypertensive and dyslipidemia conditions and weight loss. Multiple pathways mediate the vasodilator effect of *V. odorata* extract e.g., effect on Ca^2+^ and NO-mediated pathways. A pharmacologic rationale describing the use of *V. odorata* in lipid disorder treatment and hypertensive conditions exploring the phytochemicals present in *Viola* has been reported [103]. Inhibition of pancreatic lipase activity by saponins has been reported to cause a decrease in dietary fat absorption and increased fat removal in high fat diet fed mice [104]. Atherogenic index describes the risk of developing coronary heart disease, a reduction in atherogenic index was observed upon administration of the plant extract [105]. The plant extract was able to reduce some lipid profile parameters and compared favorably with the standard drug; atorvastatin, that acts by inhibiting the activity of HMG Co-A reductase [106].

### 5.6. Anti-Inflammatory Activity

*Viola diffusa* extract showed anti-inflammatory activity against dimethylbenzene-induced ear edema in ear and carrageenin-induced toe tumors in rats [107]. *Viola mandshurica* ethanolic extract significantly decreases proinflammatory cytokines and immunoglobulin E and other parameters studied in mice serum [108]. Anti-inflammatory activity of aqueous extract of *V. odorata* have been described, the mechanism may be because it can prevent inflammatory proliferation [109] because of the water soluble polysaccharides it contains [109]. The activity of *V*. *odorata* aqueous extract had prophylactic effects in formalin-induced lung damage in rats by significantly caused a decrease in lung inflammatory parameters was observed [110]. Anti-inflammatory drugs: VBHF primarily releases serotonin and histamine to prevent inflammation. This suggests that the plant may have antiasthmatic, antitussive, and bronchodilator activities. This substantiates the well-known folk uses of this plant [111]. The plant is popularly also known to be used internally and externally, for skin disorders treatment, pain, inflammation, and injuries from burn [112]. Many compounds such as *Viola*xanthin, flavonoids, saponins, alkaloids, tannins, auroxanthin, flavoxanthin, salicylic acid, and polyphenols have been reported in this plant [113]. Moreover, antioxidant flavonoid compounds, especially rutin are considered responsible for most of heartsease's biological activities of *Viola tricolor* [114]. The investigation of the topical effect of *Viola tricolor* in a model of burn injury is important and necessary to confirm its anti-inflammatory efficacy. The healing process of *Viola tricolor* gel in treating microbial infection may be due to the low pH which prevents the release of some proinflammatory cytokines [115], as well as temperature [116]. *Viola tricolor* is able to keep the gel form because of the polysaccharide [117].

### 5.7. Antipyretic Activity and Management of Pain

The possible mode of action of anti-inflammatory drugs could also be attributed to the anti-inflammatory activity displayed by antipyretic drugs such as paracetamol [118]. Yeast-induced rectal temperature of mice was significantly reduced upon intraperitoneal administration of VBME. VBME can thus be postulated to contain prostaglandin inhibitory components such as salicylic acids [119]. This antipyretic action was also demonstrated in the nonpolar fraction of *V. odorata* [120].

One defense mechanism employed by the body to combat infection is to increase the body temperature beyond the normal 36-37°C. Yeast-induced pyrexia in rabbits was stabilized with *n*-hexane, chloroform, and aqueous extract of *V. odorata* leaves. The n-hexane extract exhibited the highest antipyretic effects than the other extracts [121]. In another study, diaphoretic and febrifuge infantile disorder as well as lung trouble was managed using *V. odorata* [122] establishing the role of analgesic effects of *V. odorata*. Salicylic acid, a known sedative agent found in *V. odorata* evidently showed positive analgesic effects at 400 mg/kg aqueous and methanol extracts of *V. odorata.* The results obtained from the study compared favorably with other standard analgesic drugs examined. The analgesic effects observed in the extracts may be due to inhibition of pain response receptors or pathways leading to the production of inflammation [121].

### 5.8. Antihepatotoxic and Antinephrotoxic Activities

There was observed protective activity of methanolic extract of *V. diffusa* by preventing degeneration, necrosis, fibrosis, types I and III collagen expression, a-SMA, and TGF-b1 in the liver when induced with CCl4 [123]. Qadir et al. [124] demonstrated the hepatoprotective activity of aqueous extract of *V. odorata* against paracetamol-induced liver injury in mice. Several phytochemicals such as alkaloids, phytosterols, phenols, carotenoids, and organosulfurs have been identified from sweet violet blossom powder [103, 125, 126]. Research findings reported that these phytochemicals may be one of the factors responsible for the observed hepatoprotective role such as in the reduction of liver enzymatic activities [127], flavonoid inhibition of bile acid uptake by the hepatocytes [125], reduced bilirubin concentration upon pretreatment with flavonoids, flavonol glycoside reduction of serum AST, ALP and ALT activities, and *in vitro* flavonoids suppression of elevated levels of GOT and GPT, decreased hepatocyte damages and high antioxidant activity [128]. *Viola odorata* stem, flowers, and leaves are reportedly used in the treatment of respiratory, anti-inflammatory, tumor, urinary, liver, and kidney diseases. Animal diet supplemented with sweet violet blossom powder (SVBP) at doses of 0.2–1.6 g/100 g reportedly caused a decrease in the serum AST, ALP, and ALT activities, lipid peroxidation biomarker (malondialdehyde) levels as well as the concentration of some kidney markers such as urea and creatinine [129]. The decrease in serum concentrations of creatinine and uric acid due to feeding with plant parts of sweet violet could be adduced to the high phytochemicals' contents. Research findings reported that the possible effect of sweet violet may be due to the polyphenols content [128]. There are reports that explained that flavanone protect and maintains the kidney functionality by reducing serum urea and creatinine concentrations, excessive urination leading to loss of sodium ion and improved body weight [130, 131].

### 5.9. Insecticidal Activity

Cycloviolacin O2, O3, O8, O13, 14, 15, and 16 isolated from *V. odorata* have been identified as insecticidal agents [132]. *Viola odorata* essential oil also possesses repelling effect against culex, aedes, and anopheles mosquito strains [133]. Evidence has been shown that cyclotide kalata B1prototype possesses insecticidal activity [134]. A sixteen (16) day feeding period with kalata B1 supplement (0.825 *μ*mol/g diet) recorded a nonsurvival rate of half of the experimental *Helicoverpa punctigera* larvae population. Control and treatment diets showed a significant growth pattern difference in *H. punctigera*. Digestive enzyme inhibition by plant-based defense proteins has been proposed as a possible mode of action for the observed insecticidal activity [135]. Kalata B1 and B2 were discovered not to be responsible for the enzyme inhibitory role seen in mammalian and insect trypsins and chymotrypsins as well as *α*-amylase activity in *Helicoverpa* gut [134]. Insecticidal activity of cyclotide f-*Viola* is yet to be reported.

### 5.10. Treatment of Respiratory Disorders

Complementary system reactivation with reduced proinflammatory markers production was observed in a study of the protective role of *V. yedoensis* petroleum ether extract in the lung. The downregulation of interleukin-1b (IL-1b), interleukin-6 (IL-6), and tumor necrosis factor-a (TNF-a) together with complementary reaction alleviation could be possible for the displayed extract activity in the protection of the lungs from acute injury [136]. Cough, inflammation, pain, infections, and sleep disturbance have been reported to be effectively managed by *V. odorata*. Mucilages are protective substances that cover the mucosal membranes of the mouth, throat, and larynx, thus preventing them from various respiratory and inflammatory diseases and so on [137]. The properties of mucilage and their high amount in *V. odorata* flowers and leaves serve as emollient and demulcent, hence their use in the preparation of medicaments for respiratory and gastrointestinal diseases [138]. Climatic conditions have a large influence on the therapeutic role performed by plants. *Viola odorata* is found in cool regions and hence suitable for the treatment of heat and/or dry related ailments these include fever, cough, or respiratory infections as believed traditionally [121]. The cooling effect of *V. odorata* leaves was used internally by dioscorides (IV 121) for treating eye inflammation, heartburn, and prolapse of anus and its purple flowers externally for sore throat and epilepsy in children. Respiratory related diseases affect either the lower respiratory chamber (e.g., pneumonia) or the upper respiratory chamber (e.g., common cold, sinusitis tonsillitis, laryngitis, and pharyngitis). The disease conditions range from mild, such as common cold to severe such as asthma, allergies, sinusitis, and so on. *Viola odorata* is mainly used for skin infections, rheumatism, or urinary tract infections can also be used to cure catarrh, common cold, and cough [139].

### 5.11. Immunomodulation Activity

*Viola yedoensis*, *V. diffusa*, and *V. tricolor* aqueous extracts have been reported to exhibit significant immunomodulatory effects [48, 107]. The Violaceae, particularly *Viola tricolor,* have been noted as rich cyclotide (natural cyclic peptides) sources [90]. Cyclotides are plant compounds synthesized in the ribosomes [140], and they display unique structural cyclic backbones with three disulfide bond arrangement knotted to confer remarkable stability in them [141, 142]. Cyclotides were recently reported to block T-lymphocyte proliferation by acting as immunosuppressive peptides [143]. Peptide-containing *Oldenlandia affinis* DC. (Rubiaceae) plant extracts previously demonstrated an immunosuppressive effect towards activated human lymphocytes [144], with kalata B1 cyclotide identified as a key compound of *Oldenlandia affinis* exhibiting the immunosuppressive effect [48]. The observed immunosuppressive effects may be due to cyclotides-rich content of *V. tricolor*. Hence, the effect of *Viola* extract against proliferating cells showed the presence of cyclotides when identified using HPLC, LC-MS, and MALDI-TOF analysis. Purified kalata B1 isolated from *Oldenlandia affinis* and synthetic kalata B1 analogs were able to reduce T-cell poly functionality, stalling immune-competent cell proliferation by blocking IL-2 biology at various sites [143]. Cyclotides caused a further decrease in the expression of marker of IL-2 cell surface [143]. There is a wide range of different immunologic properties. Evidence has shown that *Viola* extract exert numerous IL-2 biologic and degranulating effects probably due to the multifunctional cyclotide components, although it did not display effect on IL-2 receptor expression [144]. The immunosuppressive properties as well as the side effects of *V. tricolor* in a low immunity individual need to be assessed [48].

### 5.12. Activity on Neurological Disorder

Animal studies have been used to evaluate the neurological effects (such as presedative and sedative) of *Viola*. Chloroform-methanolic (70 : 30) extract of *Viola* demonstrated a higher presedative and sedative effect than diazepam; the reference drug [145]. The analgesic effect of aqueous and methanolic extract of *V*. *odorata* in rats employing the tail immersion test and hot plate test showed significant effectiveness in peripheral and central models of pain [146]. Moreover, nasal drop administration of violet oil for a period of 1 month in patients with insomnia resulted in improved insomnia and sleep [147]. The numerous therapeutic roles displayed may be adduced to the various bioactive components. There are different formulations of common violet in the treatment of internal diseases, these include as pills and syrups linctus (*Laooq*; is prepared specifically for the respiratory tract) forms. To enhance effectiveness, multiple natural ingredients could be added [148]. The polyphenolic content of common violet has valuable effects on the nervous system and its potent antioxidant and neuroprotective roles [149]. The essential oil of common violet has been shown to contain a monoterpene called linalool as its main ingredient, which is shown to possess a hypnotic effect when inhaled in animal model studies, but its hypnotic effect does not affect the coordination of motor. Linalool was reported to also exhibit sedative and anticonvulsant activities in experimental mouse models [150]. Sedative effect is nontoxic to the neuron but the ethyl acetate fraction of *V. tricolor* at high concentration can cause pentobarbital hypnosis which may be due to the component(s) of the fraction [151].

### 5.13. Neurotensin Antagonism

Only one cyclotide of known neurotensin antagonistic potential has been reported, although others may not have been documented for this property. Cyclopsychotride A upon screening assay at IC_50_ of 3 *μ*M inhibited the binding of neurotensin to the cell membrane of HT-29 carcinoma in human [92]. The functional antagonistic and neurotensin-induced high HT-29 levels in the cytosol effects of Ca^2+^ were examined. An increase in intracellular concentration of Ca^2+^ in a dose-dependent fashion which was however not inhibited by any known neurotensin antagonist was seen. The reported neurotensin antagonist, cyclopsychotride A is believed to display its antagonistic activity through a different receptor [92]. The mechanism by which cyclopsychotride A increases Ca^2+^ concentrations in the cytosol of different cell lines that are nonexpressive of neurotensin receptors is yet to be reported.

## 6. Typical Doses

Three times daily intake of 2–4 g dried herb was recommended by British herbal pharmacopeia [152] and 1 g daily dose oral administration of root in physician's desk reference (PDR) [153]. *Viola odorata* has been recommended by the Iranian traditional medicine with a permissible maximum dose of 250 mg/kg containing 20 g dry flower/dose for the treatment of cough, rectal prolapse, and febrile convulsion in children. Experimental rabbits also exhibited high tolerance of the extract at an oral dose of 1.6 g/kg, no mortality was recorded [154]. Gastroenteritis, vomiting, stomachache, nervousness, and depression may be observed when the recommended dose of 5–7 g is exceeded [155]. Clinical trials of sweet violet or its interaction with supplements have not been proven, likewise its effect on pregnancy and lactation [156].

## 7. Cytotoxicity

There are no observed health hazards or side effects at the appropriate therapeutic doses [153]. Acute toxicity test after forty-eight (48) hours oral administration of 10 ml/kg of 1, 3, and 5 g/kg doses of violet leaf extract in BALC/c mice showed no toxic effect; there was no recorded lethargy, death, changes in behavior [103]. Toxicity studies after 24 hours oral administration showed that organic solvents (methanol, butanol, and n-hexane) and aqueous extracts of violet are nontoxic at 2000 mg/kg [72]. Another study revealed the cytotoxic effect of *V. tricolor* is exhibited by three cyclotides [157].

## 8. Conclusion and Future Perspective

Plants of this genus have long been used by traditional healers for the management and treatment of several human diseases including diabetes, asthma, lung diseases, fatigue, and several other diseases. The plants of the genus have also received scientific validation of their pharmacological activities including neuroprotective, immunomodulatory, antimicrobial, antiparasitic, anticancer, antihypertensive, antidyslipidemic, analgesic, antipyretic, diuretic, anti-inflammatory, anthelmintic, antioxidant, and anti-HIV. *Viola* is highly rich in different bioactive compounds and essential oils. This review harnesses some of the identified compounds as well as the pharmacological activities of *Viola*.

In addition, this review provides a guide for further studies into the pharmacokinetics of this genus. Reliable pharmacokinetic profiles in animals and humans would be crucial for a better understanding of the systemic behavior of *Viola* species. In the future, the other local uses of this genus need to be investigated; the chemical and biological relationships of these species should also be studied to expand the medicinal resources and standardize the use of *Viola* species.

## Figures and Tables

**Table 1:** Chemical constituents of some species of *Viola*.

Species	Chemical constituents	References
*Viola canescens*	Saponins, alkaloid (violin), glucosides, quercitrin, and methyl salicylate	[36–38]
*Viola odorata*	Saponins (myrosin and violin), salicylates, alkaloids, flavonoids (rutin), tannins, cycloviolacin, phenolics, coumarins, phenolic glycosides, gaultherin, violutoside (salicylic acid methyl ester), and aodoratine, 2,2,6,6-tetramethyl-4-piperidinone, violacin A, vitri peptide A, vodo peptide M & N, 2-nitroproprionic acid, mucilage, and vitamin C, cyclotides, and anthocyanins	[21, 39–42]
*Viola arvensis*	Caffeic acid, ferulic acid, and isoferulic acid	[43, 44]
*Viola brachyceras*	Cinnamic acid, ferulic acid, and neochlorogenic acid	[45]
*Viola etrusca*	Saccharides and ferulic acid	[45, 46]
*Viola tricolor*	p-Coumaric acid, cyclotides, glucose, sterols, essential oils, aromatic acids, and their derivatives	[46, 47]

**Table 2:** Summary of compounds identified in the essential oil of some *Viola* species.

S/N	Species	Part used	Total oil extracted (%)	Numbers, types, and percentage composition of secondary metabolite isolated from the oil	Identification method	References
1	*V. tricolor* L.	Fresh aerial part	97.76	8 sesquiterpenes (59.27%), 17 aliphatics (29.81%), 6 shikimic acid derivatives (8.05%), and 4 monoterpenes (0.3%), oxide (43.25%), trans-*β*-farnesene (4.01%), and bisabolol oxide A and B (7.78% and 2.28%)	GCMS	[48]
		Dried aerial parts	60.53	14 aliphatics (42.21%), 4 shikimic acid derivatives (11.20%), 2 sesquiterpenes (4.79%), and 4 monoterpenes (2.32%), hexahydrofarnesyl acetone (4.06%), methyl salicylate (1.22%), and *β*-ionone (1.00%)		

2	*V. arvensis*	Dried aerial parts	72.13	18 aliphatics (59.94%), 5 shikimic acid derivatives (8.35%), 2 monoterpenes (2.15%) and 1 sesquiterpene (1.69%) while its volatile constituents were 2-pentyl-furan (5.48%), *β*-ionone (2.09%), and hexahydrofarnesyl acetone (1.69%)	GCMS	[48]

3	*V. thianschanica*	Aerial part	—	Dibutylphthalate (15.19%), methyl hexadecanoate (8.65%), hexadecanoic acid (3.07%), and pentane-2,3-dione (2.62%)	GCMS	[49]
		Whole part		Phytol (8.61%), trimethylpentadecan-2-one (8.00%), butan-1-ol (5.48%), and docosane (5.24%)	GCMS	[50]

4	*Viola surinamensis*	Adult and plantlet leaves	—	*α*-Pinene (11.7%), *β*-pinene (5.2%), p-cymene (42.0%), trans-nerolidol (3.0%), viridiflorene (2.2%), and safrole (4.6%)	GCMS	[51]
				The plantlet leaves contained *α*-pinene (49.7%), myrcene (16.2%), terpinolene (9.9%), elemicin (11.8%), and copaene (4.6%)				

5	*V. odorata* L.	Leaves	92.77	Butyl-2-ethyl hexyl phthalate (30.10%) and 5, 6, 7, 7a-tetrahydro-4,4,7a-trimethyl-2(4H)-benzofuranone (12.03%)	HPLC-DAD and HPLC-ESI-MS	[42, 52]
		Flower	83.05	Sesquiterpene, monoterpenes, 1-phenyl butanone (22.43%), linalool (7.33%), benzyl alcohol (5.65%), *α*-cadinol (4.91%), globulol (4.32%) and viridiflorene (3.51%). Pulegone (3.33%), epi-*α*-cadinol (3.05%), terpinen-4-ol (2.31%), germacrene A (1.99%) and paramethyl anisole (1.09%), and benzyl benzoate (1.67%)	GCMS	[53]
				Other constituent include triterpene saponins, ursolic acid, transcaffeic, protocatechuic, gentisic, p-hydroxybenzoic, 4-hydroxyphenylacetic, trans-and ciscoumaric, vanillic, and salicylic acids, hex-2-enal, cis-hex-3-en-1-ol, trans-hex-3-en-1-ol, butyl acetate, oct-7-en-4-ol, 3,4-dimethylheptane, 3,7-dimethylnonane, 2,4-dimethyldodecane, 2,6,11-trimethyldodecane, 2,7,11-trimethyldodecane, hex-3-en-1-yl formate, benzyl alcohol, nona-2,6-dienal, hepta-2,5-dien-1-ol, nona-2,6-dien-1-ol, dodecan-1-ol, pentadeca-5,10-dien-1-ol, hexadec-1-ene, pentadec-3-enal, octadec-1-ene, icos-1-ene, hexadecanoic acid, and octadeca-9,12-dienoic acid	GCMS	[54, 55]

**Table 3:** Ethnomedicinal uses of *Viola.*

Species	Medicinal uses
*V. patrinii*	The flower is used treatment for cough, cold and as antipurgative, while the whole plant is used by the Chinese medical system to treat cancer [56]
*V. canescens*	The whole plant is traditionally used mostly for antipyretic, anticancer and analgesic treatment [57]
*V. serpens*	The whole plant is traditionally used as analgesic, antitumor, antihemorroids, anti-inflammatory, increased perspiration, and some respiratory diseases [58]
*V. biflora*	The whole plant is traditionally used antimicrobial agents, analgesic, antihemorroids, anti-inflammatory, increased perspiration, against constipation, and intestinal pain respiratory and skin diseases [59]
*V. arvensis*	The stem, leaves, flowers, fruits and seeds are used as against inflammation, respiratory and skin diseases, analgesic, and urinary tract infection [60]
*V. tricolor*	The stem, leaves, flowers, fruits, and seeds are used against skin conditions, cystitis, rheumatism, bronchitis and against inflammation, cough, and diuretic [60]
*V. odorata*	Fresh leaves are used for treatment of cancer, dried flowers decoction as analgesic and expectorants, seeds are used to improve removal of waste from the body, plant poultice as analgesic and against weakness [37], chewed leaves are used for anticancer, diaphoretic, febrifuge, infantile disorder, and lung troubles [61], and leaves and flowers are used for respiratory disorders [62]
*V. hondoensis*	The whole plant is used as antidiuretic, anti-inflammatory, expectorants, and for skin disease treatment [63]
*V. falconeri*	The roots are used in jaundice and flowers for cough and cold [64]
*V. cinerea*	The whole plant is used as an aphrodisiac [65]
*V. betonicifolia*	The whole plant is used as an astringent, diaphoretic, antipyretic, anticancer, purgative, and some neurological disorders [66]. The flower and leaves are used for sinusitis, skin, respiratory, and blood disorders [47], for constipation and improve waste removal [40], while the roots and fruits are used for kidney and respiratory diseases and leaves are used to treat furuncle [40]

## Data Availability

The original contributions presented in the study are included in the article. Further inquiries can be directed to the corresponding authors.
